# Iron- and Zinc-Fortified Lentil (*Lens culinaris* Medik.) Demonstrate Enhanced and Stable Iron Bioavailability After Storage

**DOI:** 10.3389/fnut.2020.614812

**Published:** 2021-01-08

**Authors:** Rajib Podder, Raymond P. Glahn, Albert Vandenberg

**Affiliations:** ^1^Department of Plant Sciences, University of Saskatchewan, Saskatoon, SK, Canada; ^2^Robert W. Holley Center for Agriculture and Health, Agricultural Research Service, United States Department of Agriculture, Ithaca, NY, United States

**Keywords:** lentil, iron, zinc, stability, dual-fortiifcation, bioavailability

## Abstract

Lentil (*Lens culinaris* Medik.) is a quick-cooking, rapidly expanding protein-rich crop with high iron (Fe) and zinc (Zn), but low bioavailability due to the presence of phytate, similar to other grains. Lentils dual fortified with Fe and Zn can significantly improve the bioavailable Fe and Zn content. Three milled lentil product types (LPTs) were fortified with Fe using NaFeEDTA [ethylenediaminetetraacetic acid iron (III) sodium salt] (Fe fortified) or Zn from ZnSO_4_·H_2_O (Zn fortified), or both (dual fortified). Fe, Zn, phytic acid (PA) concentration, and relative Fe bioavailability (RFeB%) were assessed for samples from two fortified batches (initial and for 1 year stored). Fe, Zn, and RFeB% increased significantly in two batches of samples from the three LPTs, and decreased by 5–15% after 1 year of storage. PA concentration decreased from 8 to 15% after fortification of all samples from two batches of the three LPTs but showed different patterns of influence after storage. Dual-fortified lentil fortified with 24 mg Fe and 12 mg Zn 100 g^−1^ lentil had the highest amount of Fe and Zn, and the lowest PA concentration, and RFeB% was increased from 91.3 to 519.5%. Significant (*p* ≤ 0.01) Pearson correlations were observed between Fe concentration vs. PA:Fe molar ratio (MR), Fe concentration vs. RFeB%, RFeB% vs. PA:Fe MR, and Zn concentration vs. PA:Zn MR in all samples from two batches of the three LPTs. In conclusion, dual-fortified lentil can contribute significant bioavailable Fe and Zn to populations at risk of Fe and Zn deficiency.

## Introduction

Iron (Fe) and zinc (Zn) deficiencies affect one-third and one-fifth of the world population, respectively (1). Globally, 40 and 42% of pregnant women and children, respectively, are anemic, and 20% of maternal deaths are attributed to anemia, mostly due to Fe deficiency (2, 3). Inadequate Fe intake causes the inability to maintain body temperature, increases mortality of pregnant women and newborns, decreases workability and fitness, and increases susceptibility to infectious diseases (4). Zn deficiency is also widespread, especially in lower-income countries. Globally, 17.3% of the human population has inadequate amounts of zinc, especially in South Asia (29.6%) and sub-Saharan Africa (25.6%) (5). Zinc is essential for adequate growth, immune system development, enzyme activation, protein and DNA synthesis, and neurobehavioral development (1, 5, 6). World Health Organization (WHO) and Food and Agriculture Organization (FAO) recommend 29.4 mg of Fe and 4.9 mg of Zn for males and 10.8 mg of Fe and 7 mg of Zn for females at 19–50 years of age, based on 10% bioavailability (6).

Plant-based diets are becoming more popular around the world because they provide potential health benefits by maintaining blood pressure and reducing body mass index, cholesterol level, diabetes, obesity, and cardiovascular diseases (7). Lentil is one of the most popular ingredients in plant-based diets due to its relatively quick-cooking ability, and low cost to access high-quality protein, vitamins, dietary fiber, and minerals, e.g., iron, zinc, selenium, etc. (8, 9). Lentil also is considered an excellent source of Fe (73–90 mg kg^−1^), Zn (44–54 mg kg^−1^), Se (425–673 μg kg^−1^), etc. (9, 10). The crude protein content of western Canadian lentil is 23.8 to 29.0% (11, 12).

Most cereals and legumes have significant amounts of antinutritional compounds, such as phytic acid, tannic acid, and polyphenols. These compounds inhibit the absorption of minerals, e.g., Fe and Zn (8, 13, 14). Like other cereals and legumes, lentil also has phytate phosphorus within the range of 0.08 to 0.30 g 100 g^−1^ (15). Fe and Zn can be made more bioavailable by degrading phytate in food/food products (16). Some polyphenolic compounds are degraded by enzymes during processing, thereby increasing Fe and Zn absorption (15). Plant-based Fe is non-heme, and therefore its bioavailability is comparatively lower than heme iron from animal sources (17). Plant-based Zn absorption is also lower than in animal protein–based diets due to its content of phytic acid and other inhibitors of Zn absorption (18). To overcome the problem of low bioavailability, several research organizations have developed and implemented various strategies to improve Fe and Zn concentration in food through biofortification, food fortification, supplementation, public health intervention, nutrition education, dietary diversification, and food safety measures (19).

Food fortification is one of the most widely used approaches for addressing micronutrient malnutrition. It is a sustainable approach for improving the dietary quality of targeted groups or population rapidly and preventing micronutrient deficiencies (19–21). More than 80 countries have mandatory food fortification programs for different food products based on their current nutritional status (22). Staple or partially staple foods are always recommended for fortification as a way to reach the maximum population. Several micronutrient fortified food/food products, such as wheat flour, maize flour, soy sauce, salt, edible oil, milk, and cereal, are consumed in different countries. Lentil is the most frequently consumed pulse in some countries, for example, in Bangladesh. Fortification of pulse crops is a new research area that began in 2014 at the Crop Development Center of the University of Saskatchewan, Canada, with the development of Fe-fortified lentil to address Fe deficiency in humans. More recently, a dual-fortification program to improve both Fe and Zn was initiated, and a laboratory-scale protocol was developed to fortify dehulled red and yellow lentil dal with both Fe (NaFeEDTA; ethylenediaminetetraacetic acid iron (III) sodium salt) and Zn (ZnSO_4_·H_2_O). Sensory analysis has been conducted with dual-fortified lentil to assess the consumer acceptability of lentil (23). Results from these two studies led us to assess the bioavailability of Fe using *in vitro* Caco-2 cell culture bioassay and the effect of storage period on Fe, Zn, PA concentration, and relative Fe bioavailability.

Bioavailability represents the nutrient's effectiveness and thereby determines the success or failure of micronutrient status in humans (24, 25). Assessment of bioavailability is essential for recommending any fortified food products in a diet to assess the absorbed amount of micronutrients compared with the recommended amount. Both plant-based and fortificant Fe is in the non-heme category (26), which is less bioavailable than animal sources of heme Fe. The bioavailability of Fe also depends on its solubility in the specific diet. Higher Fe concentration in food does not necessarily increase Fe bioavailability.

Several *in vitro* or *in vivo* models are used to assess Fe bioavailability, such as solubility, dializability, the gastrointestinal model, and the Caco-2 cell model (27). In this study, the Caco-2 cell bioassay was used to assess bioavailability following the model developed by Glahn et al. (28). This model combines simulated peptic and intestinal digestion followed by Fe uptake measurements using a Caco-2 cell monolayer—a human colonic adenocarcinoma (27, 28). The International Zinc Nutrition Consultation Group (IZiNCG) suggested using stunting prevalence as a biomarker to assess Zn status or deficiency (29). IZiNCG also suggested that iron-deficiency anemia is an indicator of Zn deficiency because Fe and Zn are found in the same foods, and both nutrients are hindered by similar antinutritional factors in the human body (30). Zinc bioavailability can be predicted by the Zn:phytate molar ratio (29) estimated in this study.

The expectation from any fortification program is to ensure the stability of micronutrients over time without altering the color, taste, and appearance of food (31). Exposure of fortified food to heat, light, moisture, or acidic or alkaline environments during processing and storage can affect the physico-chemical properties and stability of micronutrients (32). Any loss of micronutrient concentration or reduction of its bioavailability would impact the sustainability of a fortification program. Although several studies have reported on the stability of vitamins in fortified foods over time, the stability of minerals, such as Fe and Zn over time, is limited in fortified food/food products. It is also imperative to maintain stability during the time between the time of the food product fortification and its consumption by the end-users. A previous study showed that Fe-fortified lentil with NaFeEDTA had non-significant and minor effects on colorimetric properties after 6 months and 1 year of storage, respectively. In that study, a 1-year storage period was considered an approximate maximum storage period from processing to consumption of fortified lentil by consumers. To the best of our knowledge, this was the first study that assessed the effect of maximum storage time on Fe and Zn concentration, and on bioavailability of Fe. The present study was aimed to assess the changes of Fe, Zn, and PA concentration and the relative bioavailability of Fe over time in dual-fortified dehulled red and yellow lentil dal.

## Materials and Methods

### Preparation of Cooked Lentil Samples

Nine samples of each of the three lentil product types (LPTs)—red football (RF), red split (RS), and yellow split (YS) lentil—were used to assess Fe, Zn, and PA concentration (mg 100 g^−1^ of lentil) and relative iron bioavailability (RFeB%) (Table 1). Among the nine samples, two were unfortified control [sample 1 (unpolished) and sample 2 (polished with 0.5% canola oil)] and seven were fortified [samples 3–4 (Zn fortified), samples 5–6 (Fe fortified), and samples 7–9 (Fe and Zn fortified or dual fortified)]. In this study, NaFeEDTA and ZnSO_4_·H_2_O were used based on the results of two previous studies of the development and consumer acceptability of dual-fortified lentil (23), Podder et al., submitted. A modified dual-fortification method was used in this study to coat both Fe and Zn over the dehulled lentil surface. The combination of Fe and Zn doses for dual fortification was selected based on the Estimated Average Requirements (EAR) of micronutrients, mentioned in the WHO Fortification Guide (6) (Table 1). The target Fe concentration was higher than that of Zn concentration based on the EAR of these two minerals in humans (6). Both Fe and Zn fortificants were mixed in a similar amount of water to prepare the fortificant solution. A stand mixer (Kitchen-Aid, Artisan series 5-Quart Tilt-head, Century Avenue, Mississauga, ON, Canada) was used to mix the fortificant solution with dehulled lentils followed by a polish application of 0.5% canola oil. After 10 min of mixing in the bowl, fortified lentil was poured into a round aluminum foil tray placed over a Barnstead Thermolyne M49235 Bigger Bill Orbital Shaker (Sigma-Aldrich Corp., St. Louis, MO, USA). A 250-W electric heat lamp (NOMA incandescent, clear, 130 V heat lamp; Trileaf Distributors, Toronto, ON, Canada) and a mini portable desk fan (model 043-5498-4; Trileaf Distributors) were used to provide both heat and air to achieve the desired moisture content (<14%) of the lentil products. Moisture content and water activity of the fortified samples were determined at the Saskatchewan Food Industry Development Center, following the “Official Methods of Analysis of the Association of Official Analytical Chemists (AOAC),” 16th Edition (1995) 42.1.03 (978.18) (33).

**Table 1:** Nine milled lentil samples from each of the three lentil product types (red football, red split, and yellow split) used for dual fortification with different doses of Fe and Zn from NaFeEDTA and ZnSO_4_·H_2_O, respectively.

**Samples**	**Fortification status**	**Fortificant dose/s added 100 g**^****−1****^ **of lentil**	
		**Fe (mg) NaFeEDTA**	**Zn (mg) ZnSO_4_·H_**2**_O**
Sample 1	Control	Unfortified and unpolished	
Sample 2	Control	Unfortified and polished with 0.5% canola oil	
Sample 3	Zn fortified (single)	–	6
Sample 4	Zn fortified (single)	–	12
Sample 5	Fe fortified (single)	16	–
Sample 6	Fe fortified (single)	24	–
Sample 7	Fe and Zn fortified (dual)	12	12
Sample 8	Fe and Zn fortified (dual)	16	8
Sample 9	Fe and Zn fortified (dual)	24	12

Half of the lentils from each sample were stored separately in clear plastic bags (Ronco, Toronto, ON, Canada), similar to methods traditionally used to store dal products. The 1-year storage period was considered an approximate maximum storage period from processing to consumption by dal consumers. The other half of the lentil samples were cooked to prepare a soup according to a traditional Bangladeshi recipe where lentil, deionized water, canola oil, salt, turmeric powder, and onion were used as ingredients in a 15:70:4:3:2:6 ratio by weight (34). All foods were cooked with 18 MΩ deionized water, and stainless-steel cookware was used to avoid contamination of additional Fe and Zn. Prepared soup samples were cooled at room temperature for 2 h, frozen at −80°C for 24 h, then freeze dried using a FreeZone 12 L Console Freeze Dry System with Stoppering Tray Dryers (Labconco, Model 7759040, Kansas City, MO, USA) for 72 h, and then stored at room temperature (35). A 10-g sample of each freeze-dried cooked sample was finely ground and sent to the USDA-ARS Robert Holley Center for Agriculture and Health (Ithaca, NY, USA) to determine Fe, Zn, and PA concentration. From the 10-g sample, 0.5 g of each of the three repetitions was used in the Caco-2 cell bioassay to estimate the RFeB% (35, 36).

### Assessment of Fe, Zn, and PA Concentration and RFeB%

The concentrations of Fe and Zn of all three LPTs were quantified with an inductively coupled argon plasma emission spectrometer (iCAP 6500 series; Thermo Jarrell Ash Corp., Franklin, MA, USA) following the procedure of Glahn et al. (37). RFeB% for all three LPTs samples were assessed using the Caco-2 cell bioassay to estimate cell ferritin formation as the measure of cell Fe uptake and bioavailability (38–40). The bioavailability assessment was conducted for three replicates of each cooked lentil sample. The Caco-2 cell bioassay for Fe bioavailability has been extensively published by Glahn, with little change in the original conditions published in 1998 (39). The model utilizes simulated gastric and intestinal digestion conditions whereby the intestinal digestion occurs simultaneously with the opportunity for Fe uptake, thus replicating the physiology of the upper small intestine.

Ferritin formation by Caco-2 cell monolayers has proven to be highly sensitive and accurately measures food Fe availability in this *in vitro* system. Caco-2 cell monolayers (American Type Culture Collection, Rockville, MD) were seeded at a density of 50,000 cells cm^−2^ in collagen-treated six-well plates (Costar Corp., Cambridge, MA). The cells were then grown in Dulbecco's Modified Eagle Medium (GIBCO, Grand Island, NY) with 10% *v*/*v* fetal calf serum (GIBCO), 25 mmol/L HEPES, and 1% antibiotic antimitotic solution (GIBCO) and placed in an incubator for 13 days, then used in the Fe uptake experiments. Each lentil sample (0.5 g) was digested in an *in vitro* digestion system using a digestion solution (pepsin, pancreatin, and bile extract) at pH 7.0, and the digest was referred to as “intestinal digest.”

Before the intestinal digestion, growth medium was removed from each culture well, and the cell culture was washed twice with 37°C Minimum Essential Media (MEM, no. 41500; GIBCO, Inc.) at pH 7.0. Then the six-well culture plates with cell monolayers were prepared to complete the intestinal digestion described by Glahn et al. (37). The intestinal digest cell monolayers were then harvested for ferritin analysis at 24 h after the start of the intestinal digestion period. The medium covering the cells was removed, and the cells were harvested and washed once with a 2-ml volume of a “rinse” solution containing 140 mmol/L NaCl, 5 mmol/L KCl, and 10 mmol of PIPES, at pH 7. After rinsing, 2 ml of deionized water was placed on each monolayer. The plates were then placed on a rack with the bottom of each plate in contact with the water of a benchtop sonicator (Lab-Line Instruments, Melrose Park, IL), which was kept in a cold room at 4°C. The cells were sonicated for 15 min and then scraped from the plate surface and harvested along with the 2-ml volume of water in each well. The samples were immediately frozen and stored at −20°C. Caco-2 cell protein was measured from samples that had been solubilized in 0.5 mol/L NaOH, using a semi-micro adaptation of the Bio-Rad DC protein assay kit (Bio-Rad Laboratories, Hercules, CA). A one-stage, two-site immunoradiometric assay was used to measure Caco-2 cell ferritin content (FER-Iron II Ferritin Assay; RAMCO Laboratories, Houston, TX). A 10-μl sample of the sonicated Caco-2 cell monolayer, harvested in 2 ml of water, was used for each ferritin measurement. Analysis of the Fe in solutions and digested biological samples was determined by inductively coupled argon plasma emission spectrometry (ICAP model 61E trace analyzer; Thermo Jarrell Ash Corp).

Ferritin values of the fortified lentil samples were compared with the control samples (sample 1, unfortified and unpolished) for each of the three LPTs to calculate the RFeB%, using the following equation: relative Fe bioavailability (RFeB%) = [(ng ferritin of the lentil sample/mg protein of the lentil sample)/(ng ferritin/mg protein of the control lentil)] × 100 (38). The calculated relative Fe bioavailability (RFeB%) is used to assess the percent increase or decrease of bioavailability compared with the control. PA content was measured as phosphorous released by phytase and alkaline phosphatase via a colorimetric assay kit (K-PHYT 12/12; Megazyme International, Wicklow, Ireland) (37).

### Prediction of Zn Bioavailability Using PA:Zn Ratio

Evidence from the literature showed that Zn delivery is very much related to its concentration, and low PA:Zn ratio might help predict the Zn bioavailability (27, 29). Dietary Zn is mainly absorbed in the duodenum by active and passive mechanisms, and unlike Fe, a sensitive biomarker of Zn status is yet to be identified (29). In this study, Zn bioavailability was not measured, but the phytate:Zn molar ratio (MR) was used to analyze the same samples that were assessed for Fe bioavailability.

### Data Analysis

Data were analyzed using SAS version 9.4 (SAS Institute Inc., Cary, NC, USA). Differences in Fe concentration, Zn concentration, RFeB%, and PA concentration among the nine samples from each of the three LPTs were verified using one-way ANOVA. Data were expressed as mean ± SD for Fe, Zn, and PA concentration. Paired *t*-test analysis calculated the effect of storage period on all variables. Fisher's least significant difference was calculated considering a significance level of 5% (*p* < 0.05). Pearson correlations (*p* < 0.05) among Fe and Zn concentration, RFeB%, and PA concentration were calculated (38). Fe and Zn concentration, RFeB%, PA, PA:Fe molar ratio (MR), and PA:Zn MR were compared to assess the effect of Fe and Zn fortificants on fortified and unfortified lentil samples.

## Results

### Effects of Fortification and Storage Period on Fe Concentration of Three LPTs

The mean Fe concentration of nine lentil samples of the three LPTs from two batches (initial and 1 year of storage) is shown in Table 2. Within each batch, non-significant differences in Fe concentration were observed between the two controls (untreated vs. those treated with 0.5% canola oil) and two Zn-fortified samples (samples 3 and 4) of all three LPTs. The remaining five samples (samples 5–9) had significantly different Fe concentrations than the control for all three LPTs in both batches. Two Fe-fortified samples (samples 5 and 6) had significant differences for Fe concentration with the Fe dose increase from 16 to 24 mg 100 g^−1^ of lentil. The three dual-fortified samples (samples 7–9) were significantly different for Fe concentration with an increment of fortificant Fe concentration from 12 to 24 mg Fe 100 g^−1^ of lentil. Among the three LPTs in two batches, the highest amount of Fe was found in dual-fortified lentil fortified with 24 mg of Fe and 12 mg of Zn 100 g^−1^ of lentil, and the lowest amount of Fe was found in unfortified control and two Zn-fortified samples.

**Table 2:** Iron concentration (mg 100 g^−1^ of lentil) of nine milled lentil samples from each of the three product types (red football, red split, and yellow split) containing unfortified lentil (samples 1–2) and fortified lentil (samples 3–9) assessed using inductively coupled argon-plasma emission spectrometer.

**Samples**	**^a^Average Fe concentration (mg 100 g^****−1****^**of lentil)****								
	**Red football**			**Red split**			**Yellow split**		
	**Initial**	**After 1 year**	**Fe (%) change**	**Initial**	**After 1 year**	**Fe (%) change**	**Initial**	**After 1 year**	**Fe (%) change**
Sample 1^b^	7.5 ± 0.2A	6.9 ± 0.9A	−8.0^*^	7.1 ± 0.8A	6.6 ± 0.3A	−7.6^*^	5.9 ± 0.2A	5.2 ± 0.2B	−10.7^*^
Sample 2^c^	7.6 ± 1.4A	6.9 ± 0.3A	−8.8^*^	7.3 ± 0.5A	6.4 ± 0.5A	−11.6^*^	5.9 ± 0.6A	5.1 ± 0.3A	−12.3^*^
Sample 3^d^	7.5 ± 0.4A	6.7 ± 0.9A	−9.9^*^	7.4 ± 0.5A	6.5 ± 0.9A	−12.9^*^	6.0 ± 0.4A	5.2 ± 0.3B	−12.2^*^
Sample 4^d^	7.7 ± 0.7A	6.8 ± 0.9A	−11.7^*^	7.4 ± 0.4A	6.5 ± 1.7A	−11.6^*^	6.0 ± 0.7A	5.2 ± 0.1A	−14.1^*^
Sample 5^e^	25.7 ± 6.9C	23.7 ± 0.2C	−8.0^*^	20.5 ± 0.4B	18.1 ± 0.1 B	−11.8^*^	19.6 ± 2.4B	17.0 ± 0.1C	−13.3^*^
Sample 6^e^	27.2 ± 0.7D	24.4 ± 0.3D	−10.4^*^	31.1 ± 3.5D	27.8 ± 0.4D	−10.8^*^	21.6 ± 1.1C	18.9 ± 0.1C	−12.5^*^
Sample 7^f^	20.5 ± 4.9B	18.0 ± 0.6B	−12.1^*^	20.8 ± 3.1B	18.1 ± 0.2B	−13.4^*^	19.4 ± 0.2B	18.1 ± 2.9C	−6.4^*^
Sample 8^f^	27.1 ± 4.3D	24.5 ± 1.0D,E	−9.6^*^	27.9 ± 4.6C	25.0 ± 2.9C	−10.7^*^	29.9 ± 2.5D	26.3 ± 0.4D	−11.9^*^
Sample 9^f^	28.6 ± 0.6E	24.9 ± 0.5E	−13.2^*^	31.6 ± 2.8D	28.5 ± 0.4D	−9.9^*^	32.9 ± 2.2E	30.0 ± 1.3E	−8.7^*^
Pearson correlation^g^	0.99^**^			0.99^**^			0.99^**^		

Different roman letters within each column represent significant differences (p < 0.05) of Fe concentration between nine samples. “^*^” represents significant differences (p < 0.05) of Fe concentrations among the two batches (initial or 1 year of storage) and within each product type.

a *Mean ± SD. Mean scores for Fe concentration followed by different letters within columns are significantly different (p < 0.001)*.

b *Unfortified control lentil*.

c *Unfortified control but polished with 0.5% canola oil*.

d *Zn-fortified lentil with ZnSO_4_·H_2_O*.

e *Fe-fortified lentil with NaFeEDTA*.

f *Dual-fortified lentil with NaFeEDTA and ZnSO_4_·H_2_O*.

g *Pearson correlation coefficients for Fe concentration between two batches*.

** *Correlation is significant at the 0.01 level (2-tailed)*.

* *Correlation is significant at the 0.05 level (2-tailed)*.

For all three LPTs, Fe concentration decreased significantly in both fortified and unfortified samples after storage for 1 year (Table 2). In RF, RS, and YS lentil samples, Fe concentration decreased by 8 to 14.5%, 7.6 to 13.4%, and 6.4 to 14.1%, respectively.

### Effect of Fortification and Storage Period on Zn Concentration of Three LPTs

The average Zn concentrations of the nine samples of RF, RS, and YS lentils from two batches (initial and 1 year of storage) are shown in Table 3. A non-significant difference for Zn concentration was observed between the two controls (untreated vs. treated with 0.5% canola oil) and the two Fe-fortified (samples 5 and 6) samples for all three LPTs within two batches. The remaining five samples (samples 3–4, 7–9) had significantly different Zn concentrations compared with controls for all three LPTs in two batches. Two Zn-fortified samples (samples 3–4) and three dual-fortified samples (samples 7–9) were significantly different for Zn concentration with an increment of the fortificant (ZnSO_4_·H_2_O) Zn concentration from 8 to 12 mg Zn 100 g^−1^ of lentil. Among the three LPTs from two batches, the highest amount of Zn was found in dual-fortified lentil fortified with 24 mg of Fe and 12 mg of Zn 100 g^−1^. The lowest amount of Zn was found in the unfortified control and two Fe-fortified samples for all three LPTs and two batches.

**Table 3:** Zinc concentration (mg 100 g^−1^ of lentil) of nine milled lentil samples of each of the three product types (red football, red split, and yellow split) containing unfortified lentil (samples 1–2) and fortified lentil (samples 3–9) assessed using inductively coupled argon-plasma emission spectrometer.

**Samples**	**^a^**Average Zn concentration (mg 100 g**^****−1****^**of lentil)****								
	**Red football**			**Red split**			**Yellow split**		
	**Initial**	**After 1 year**	**Zn (%) change**	**Initial**	**After 1 year**	**Zn (%) change**	**Initial**	**After 1 year**	**Zn (%) change**
Sample 1^b^	4.3 ± 0.2A	3.1 ± 0.2A	−8.7^*^	4.4 ± 0.6A	2.9 ± 0.2B	−11.0^*^	3.9 ± 0.5A	2.6 ± 0.1A	−15.0^*^
Sample 2^c^	4.3 ± 0.3A	3.1 ± 0.1A	−9.4^*^	4.3 ± 0.4A	2.9 ± 0.5A,B	−13.4^*^	3.9 ± 0.2A	2.6 ± 0.2A	−16.7^*^
Sample 3^d^	9.9 ± 0.4C	9.2 ± 0.2B	−10.7^*^	9.8 ± 0.1B	8.5 ± 0.1C	−14.7^*^	8.8 ± 0.5B	7.6 ± 0.2B	−16.4^*^
Sample 4^d^	15.5 ± 0.8E	14.2 ± 0.8C	−10.5^*^	15.2 ± 0.3E	13.0 ± 0.3D	−13.7^*^	13.9 ± 0.5E	12.1 ± 0.1C	−15.4^*^
Sample 5^e^	4.4 ± 0.1AB	3.2 ± 0.2A	−11.3^*^	4.3 ± 2.9A	2.9 ± 0.5A	−14.7^*^	3.9 ± 0.1A	2.7 ± 0.1A	−17.3^*^
Sample 6^e^	4.5 ± 0.2B	3.2 ± 0.1A	−12.0^*^	4.4 ± 3.3A	2.9 ± 1.1A	−15.5^*^	3.9 ± 0.2A	2.6 ± 0.1B	−17.7^*^
Sample 7^f^	15.7 ± 0.8E,F	16.3 ± 1.3F	10.9^*^	15.3 ± 1.9E	14.9 ± 4.3F	12.9^*^	13.9 ± 0.3F	14.8 ± 1.6D	9.0^*^
Sample 8^f^	13.9 ± 0.5D	14.6 ± 0.8E	12.6^*^	13.4 ± 1.2C	13.7 ± 0.6D	4.5^*^	12.1 ± 0.6C	13.1 ± 0.3C	10.2^*^
Sample 9^f^	15.0 ± 0.6E	16.9 ± 0.6G	11.9^*^	14.6 ± 1.3D	15.3 ± 2.1E	11.1^*^	13.3 ± 0.3D	15.3 ± 0.7D	9.4^*^
Pearson correlation^g^	0.99^**^			0.99^**^			0.99^**^		

Different roman letters within each column represent significant differences (p < 0.05) of Zn concentration between nine samples. “^*^” represents significant differences (p < 0.05) of Zn concentrations among the two batches (initial or 1 year of storage) and within each product type.

a *Mean ± SD. Mean scores for Zn concentration followed by different roman letters within columns are significantly different (p < 0.001)*.

b *Unfortified control lentil*.

c *Unfortified control but polished with 0.5% canola oil*.

d *Zn-fortified lentil with ZnSO_4_·H_2_O*.

e *Fe-fortified lentil with NaFeEDTA*.

f *Dual-fortified lentil with NaFeEDTA and ZnSO_4_·H_2_O*.

g *Pearson correlation coefficients for Zn concentration between two batches*.

** *Correlation is significant at the 0.01 level (2-tailed)*.

* *Correlation is significant at the 0.05 level (2-tailed)*.

Unlike Fe concentration, in all three LPTs, Zn concentration was decreased significantly in single fortified and unfortified lentil samples after storage for 1 year (Table 3). In contrast, the average Zn concentration was increased significantly in three dual-fortified samples from all three LPTs after 1 year of storage. In three dual-fortified samples of RF, RS, and YS lentils, Zn concentration was increased by 10.0 to 12.6%, 4.5 to 12.9%, and 9.0 to 10.2%, respectively.

### Effect of Fortification and Storage Period on PA Concentration of Three LPTs

The average phytic acid (PA) concentration of nine lentil samples of each of the three LPTs is shown in Table 4. In RF lentil samples from two batches, PA concentration was reduced significantly from control (unpolished) lentil after fortification, and dual-fortified lentil samples had the lowest PA concentration. In RS samples, a non-significant difference was observed for PA concentration between two controls and the Zn-fortified samples, and the lowest amount of PA was observed in dual-fortified sample 9 and Fe-fortified sample 6. For YS lentil, the unfortified and polished sample (sample 2) had the highest PA concentration, significantly more than other samples in both batches. The lowest PA was observed in the dual-fortified sample 9.

**Table 4:** Phytic acid (PA) concentration (mg 100 g^−1^ of lentil) of nine milled lentil samples from each of the three product types (red football, red split, and yellow split) containing unfortified lentil (samples 1–2) and fortified lentil (samples 3–9) assessed using inductively coupled argon-plasma emission spectrometer.

**Samples**	**^a^**Average PA concentration (mg 100 g**^****−1****^**of lentil)****								
	**Red football**			**Red split**			**Yellow split**		
	**Initial**	**After 1 year**	**PA (%) change**	**Initial**	**After 1 year**	**PA (%) change**	**Initial**	**After 1 year**	**PA (%) change**
Sample 1^b^	0.65 ± 0.1A	0.66 ± 0.1A	0.8	0.82 ± 0.0A	0.78 ± 0.1A	−4.8^*^	0.89 ± 0.2B,C	0.80 ± 0.1D,E	−10.2^*^
Sample 2^c^	0.63 ± 0.1A,B	0.64 ± 0.0B	0.5	0.83 ± 0.0A	0.81 ± 0.1A	−2.2	0.96 ± 0.1A	0.87 ± 0.2A	−9.2^*^
Sample 3^d^	0.62 ± 0.1B	0.65 ± 0.0A,B	3.7	0.83 ± 0.0A	0.81 ± 0.0A	−2.1	0.92 ± 0.0B	0.88 ± 0.2A	−4.7^*^
Sample 4^d^	0.62 ± 0.1B	0.64 ± 0.1A,B	2.9^*^	0.80 ± 0.1A	0.79 ± 0.0A	−1.7	0.92 ± 0.0B	0.83 ± 0.1CD	−9.8^*^
Sample 5^e^	0.52 ± 0.0D	0.57 ± 0.0D,C	11.1^*^	0.75 ± 0.1B	0.76 ± 0.0A	1.5	0.87 ± 0.1C	0.80 ± 0.1D,E	−8.2^*^
Sample 6^e^	0.55 ± 0.0C	0.60 ± 0.1C	9.8^*^	0.71 ± 0.0C	0.70 ± 0.1B	−0.9	0.87 ± 0.0C	0.79 ± 0.0D,E	−8.4^*^
Sample 7^f^	0.56 ± 0.1C	0.56 ± 0.0E	1.1	0.75 ± 0.1B	0.76 ± 0.2A	−2.1	0.87 ± 0.0C	0.86 ± 0.1A,B	−1.9
Sample 8^f^	0.51 ± 0.1D	0.58 ± 0.0D	13.3^*^	0.73 ± 0.2B,C	0.76 ± 0.1A	−3.2	0.79 ± 0.0D	0.78 ± 0.0E	−1.0
Sample 9^f^	0.53 ± 0.0D	0.60 ± 0.0C	13.4^*^	0.71 ± 0.1C	0.69 ± 0.0B	2.7	0.79 ± 0.1D	0.83 ± 0.0B	5.2
Pearson correlation^g^	0.90^**^			0.91^**^			0.68^**^		

Different roman letters within each column represent significant differences (p < 0.05) of PA concentration between nine samples. “^*^” represents significant differences (p < 0.05) of PA concentrations among the two batches (initial or 1 year of storage) and within each product type.

a *Mean ± SD. Mean scores for PA concentration followed by different letters within columns are significantly different (p < 0.001)*.

b *Unfortified control lentil*.

c *Unfortified control but polished with 0.5% canola oil*.

d *Zn-fortified lentil with ZnSO_4_·H_2_O*.

e *Fe-fortified lentil with NaFeEDTA*.

f *Dual-fortified lentil with NaFeEDTA and ZnSO_4_·H_2_O*.

g *Pearson correlation coefficients for PA concentration between two batches*.

** *Correlation is significant at the 0.01 level (2-tailed)*.

* *Correlation is significant at the 0.05 level (2-tailed)*.

The effect of storage period on PA concentration in all three LPTs is shown in Table 4. In RF lentil, non-significant differences were observed for PA concentration between the two batches for samples 1–3, whereas PA concentration increased significantly in five samples (samples 4–9) after 1 year of storage. Unlike RF samples, after 1 year of storage, significant differences were observed for PA concentration for samples 1–3, whereas non-significant differences were observed for samples 4–9. For YS lentil, all three dual-fortified lentils had similar PA concentrations after 1 year of storage. The remaining six samples (samples 1–6) had significantly different PA concentrations compared with the initial batch lentil samples. Overall, three LPTs showed a different pattern of influence of storage time on PA concentration.

### Iron Bioavailability Assessment of Unfortified and Fortified Lentil

The bioavailability of iron was assessed using Caco-2 cell bioassay on three replicates of each lentil sample. Ferritin [“ng ferritin (mg protein)^−1^”] values from control (sample 2) and fortified lentil samples (samples 3–9) were compared with the control lentil (untreated; sample 1, Fe concentration of 74 μg g^−1^) to calculate the relative iron bioavailability (RFeB%) following the formula mentioned in the Materials and methods section. The resulting index of relative Fe bioavailability (RFeB%) was used hereafter.

**Figure 1:**
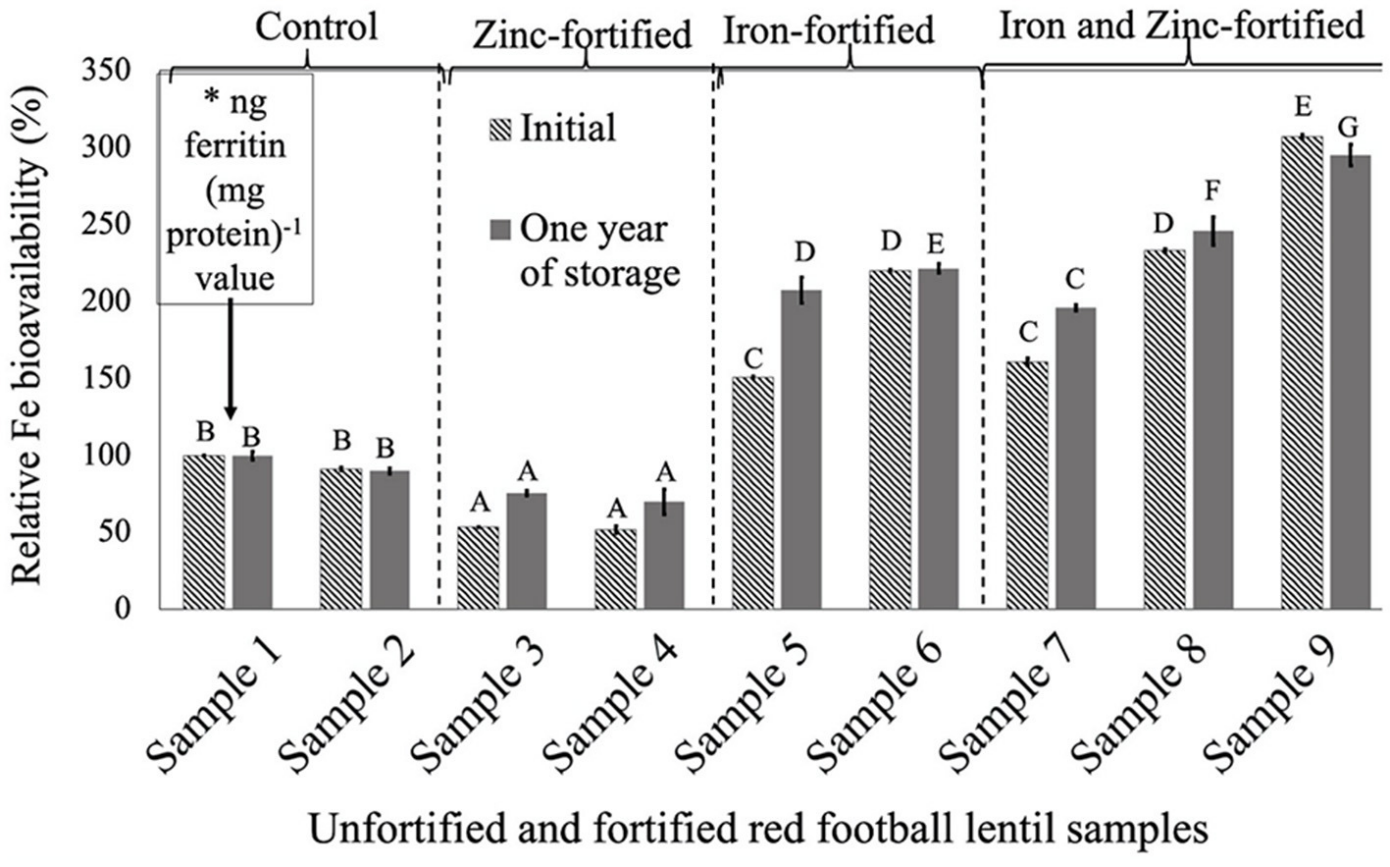
Relative iron bioavailability (RFeB%) of nine dehulled red football lentil samples, [unfortified and unpolished (sample 1), unfortified and polished with 0.05% canola oil (sample 2), fortified with 6 mg Zn 100 g^−1^ lentil (sample 3), fortified with 12 mg Zn 100 g^−1^ lentil (sample 4), fortified with 16 mg Fe 100 g^−1^ lentil (sample 5), fortified with 24 mg Fe 100 g^−1^ lentil (sample 6), fortified with 12 mg Zn and 12 mg Fe 100 g^−1^ lentil (sample 7), fortified with 8 mg Zn and 16 mg Fe 100^−1^ lentil (sample 8), and fortified with 12 mg Zn and 24 mg Fe 100 g^−1^ lentil (sample 9)] assessed using Caco-2 cell bioassay. (*) ng ferritin (mg protein)^−1^ value (11.8 ± 0.8, initial, and 67.1 ± 4.6, 1-year storage) of unfortified and unpolished control sample (sample 1) was used to calculate the relative Fe bioavailability (%) of other samples (samples 2 to 9). Different roman letters above each patterned and solid gray bar are significantly different RFeB% at initial and after 1 year of storage, respectively.

**Figure 2:**
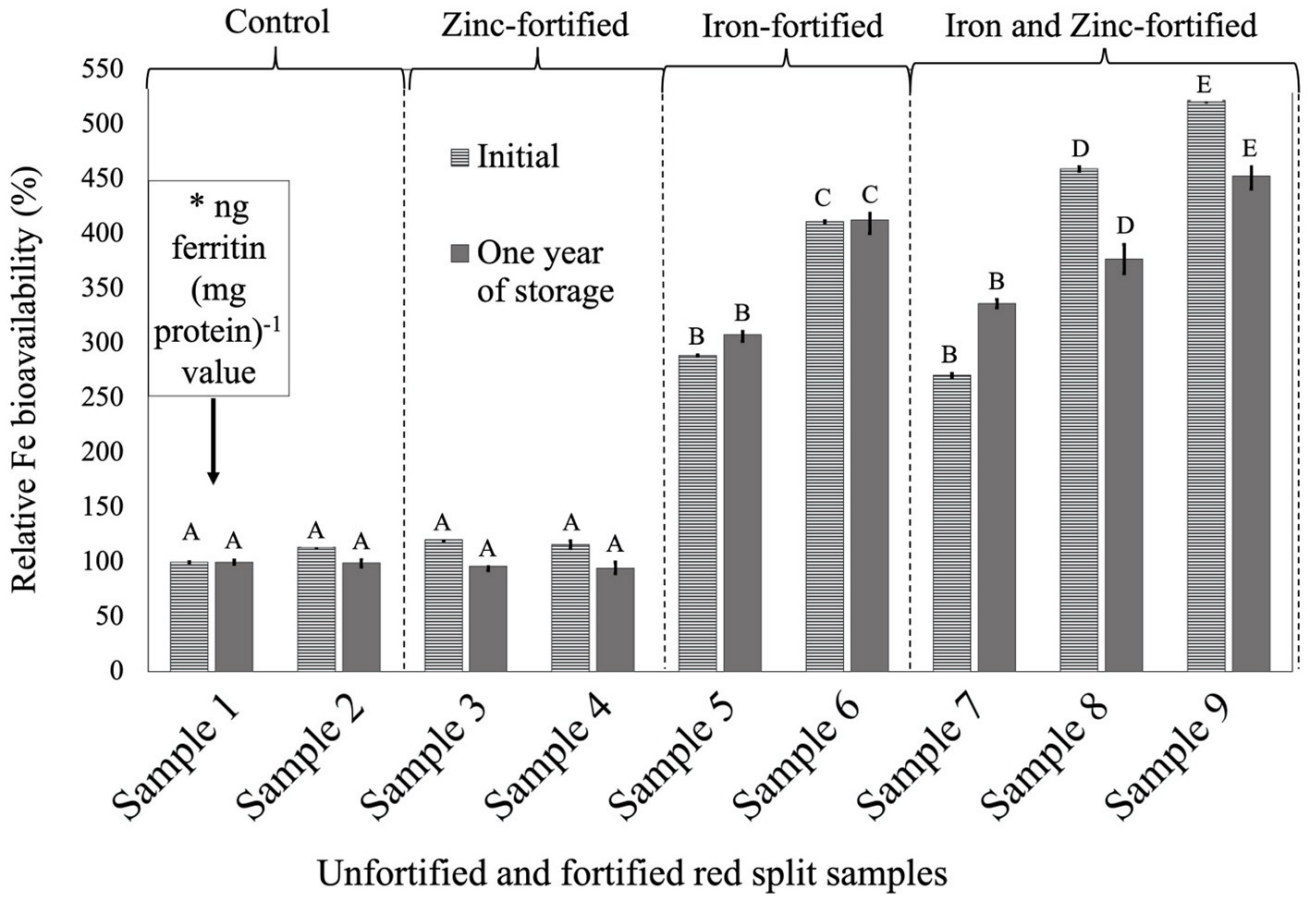
Relative iron bioavailability (RFeB%) of nine dehulled red split lentil samples, [unfortified and unpolished (sample 1), unfortified and polished with 0.05% canola oil (sample 2), fortified with 6 mg Zn 100 g^−1^ lentil (sample 3), fortified with 12 mg Zn 100 g^−1^ lentil (sample 4), fortified with 16 mg Fe 100 g^−1^ lentil (sample 5), fortified with 24 mg Fe 100 g^−1^ lentil (sample 6), fortified with 12 mg Zn and 12 mg Fe 100 g^−1^ lentil (sample 7), fortified with 8 mg Zn and 16 mg Fe 100 g^−1^ lentil (sample 8), and fortified with 12 mg Zn and 24 mg Fe 100 g^−1^ lentil (sample 9)] assessed using Caco-2 cell bioassay. (*) ng ferritin (mg protein)^−1^ value (7.1 ± 0.7, initial, and 46.4 ± 0.2, 1-year storage) of unfortified and unpolished control sample (sample 1) was used to calculate the relative Fe bioavailability (%) of other samples (samples 2 to 9). Different roman letters above each patterned and solid gray bar are significantly different RFeB% at initial, and after 1 year of storage, respectively.

**Figure 3:**
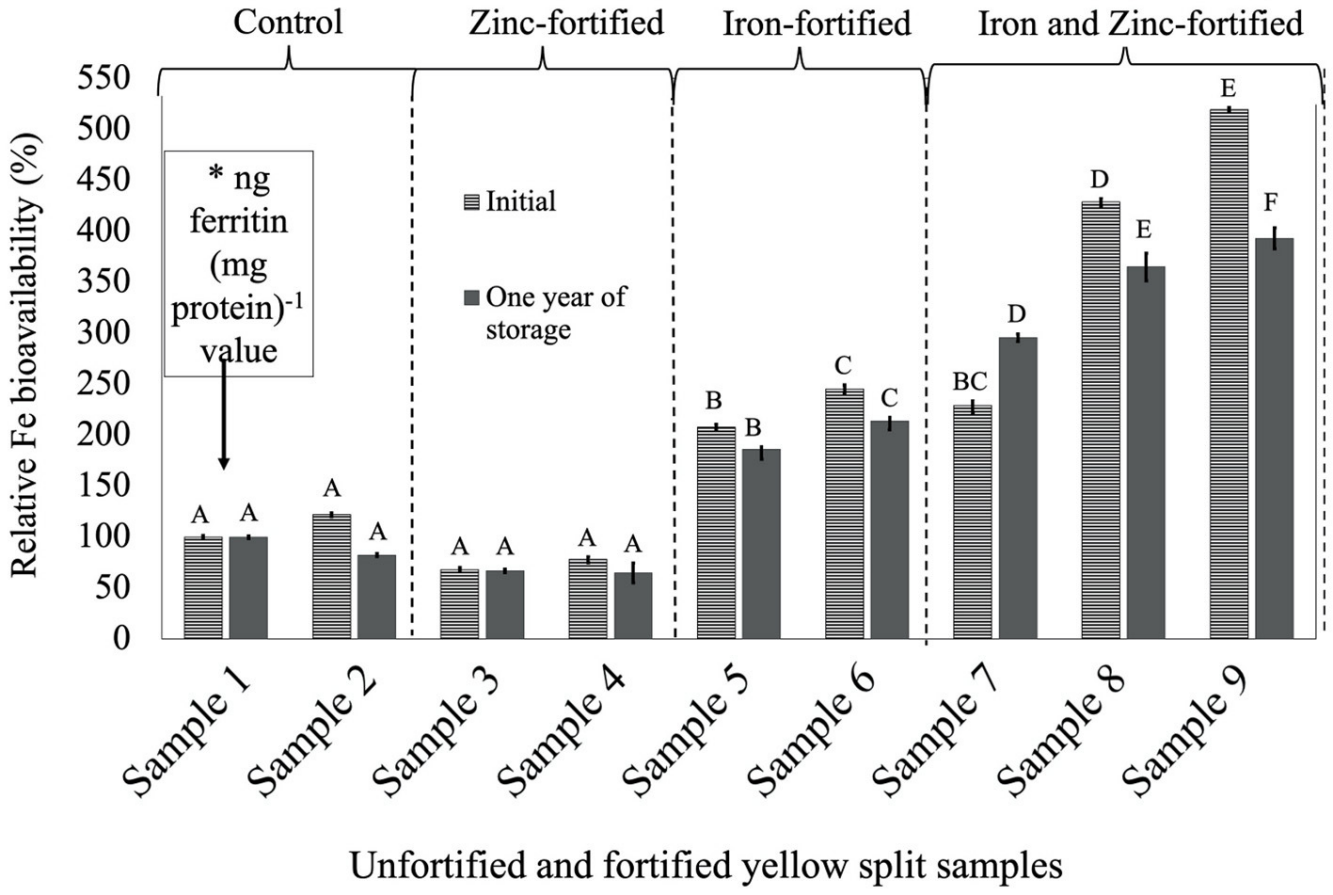
Relative iron bioavailability (RFeB%) of nine dehulled yellow split lentil samples, [unfortified and unpolished (sample 1), unfortified and polished with 0.05% canola oil (sample 2), fortified with 6 mg Zn 100 g^−1^ lentil (sample 3), fortified with 12 mg Zn 100 g^−1^ lentil (sample 4), fortified with 16 mg Fe 100 g^−1^ lentil (sample 5), fortified with 24 mg Fe 100 g^−1^ lentil (sample 6), fortified with 12 mg Zn and 12 mg Fe 100 g^−1^ lentil (sample 7), fortified with 8 mg Zn and 16 mg Fe 100 g^−1^ lentil (sample 8), and fortified with 12 mg Zn and 24 mg Fe 100 g^−1^ lentil (sample 9)] assessed using Caco-2 cell bioassay. (*) ng ferritin (mg protein)^−1^ value (7.1 ± 0.7, initial, and 44.9 ± 1.3, 1-year storage) of unfortified and unpolished control sample (sample 1) was used to calculate the relative Fe bioavailability (%) of other samples (samples 2 to 9). Different roman letters above each patterned and solid gray bar are significantly different RFeB% at initial and after 1 year of storage, respectively.

For all three LPTs, RFeB% was increased with the increase of Fe concentration but with a different pattern (Figures 1–3). In RF lentil samples, significant differences were observed between control and fortified samples for ferritin concentration. The RFeB% ranged from 51.7% (sample 4) to 307.3% (sample 9) in the initial batch and 69.2% (sample 4) to 295.3% (sample 9) in samples from 1 year of storage (Supplementary Table 1). The control lentil (sample 2) had an RFeB% value of 91.3 and 90.1% in the initial and the 1-year stored samples, respectively. Within both batches, non-significant differences were observed for ferritin concentration between the control samples. Two Zn-fortified samples (samples 3–4) had significantly similar ferritin concentration, at the initial stage, but 46.7 and 48.3% less RFeB% respectively than the control (sample 2). Similar results were observed for the 1-year stored samples, but with a different rate (Supplementary Table 1). Again, for both batches, in two Fe-fortified (samples 5 and 6) and three dual-fortified samples (samples 7–9), ferritin concentration and RFeB% increased significantly with the increment of Fe doses from the NaFeEDTA fortificant.

In RS lentil samples, significant differences were observed between control and fortified samples for ferritin concentration. The RFeB% ranged from 113.6% (sample 4) to 521.8% (sample 9) for the initial batch, and 94.6% (sample 4) to 452.9% (sample 9) in 1-year stored samples (Supplementary Table 2). The control (sample 2) sample had an RFeB% value of 113.6 and 99.4% for initial and 1-year stored samples, respectively. Within both batches, non-significant differences were observed for ferritin concentration between control samples. Two Zn-fortified samples (samples 3–4) had only 20.6 and 16.0% higher RFeB%, respectively, than the control initially. Samples 3 and 4 had less RFeB% than the control after 1 year of storage (Supplementary Table 2). Again, similar to RF lentil samples for both batches, in two Fe-fortified (samples 5 and 6) and three dual-fortified samples (samples 7–9), ferritin concentration and RFeB% increased significantly with the increment of Fe doses from the NaFeEDTA fortificant.

In YS lentil, like the other two LPTs, significant differences were observed for ferritin concentration and RFeB%, ranging from 68.3% (sample 4) to 519.5% (sample 9) in the initial batch and 65.0% (sample 4) to 393.3% (sample 9) in batch 2 (Supplementary Table 3). The control (sample 2) lentil had an RFeB% value of 122.0 and 82.4% in the initial and 1-year stored batches, respectively. Within both batches, non-significant differences were observed for ferritin concentration between the two control samples. In the initial batch, two Zn-fortified samples (samples 3 and 4) had 31.7 and 21.8% less RFeB%, respectively, compared with the control (sample 1). Similar results were observed for lentil samples stored for 1 year, but with different rates (Supplementary Table 3). In both batches, ferritin concentration and RFeB% were significantly increased in two Fe-fortified (samples 5 and 6) and three dual-fortified samples (samples 7–9), with an increase of Fe-fortificant dose.

The overall results showed that Zn fortification might not affect or influence the RFeB% in dual-fortified lentil samples for all three LPTs but was decreased when lentil was fortified with ZnSO_4_.H_2_O only.

### PA:Fe and PA:Zn MR of Initial and 1-Year Stored Lentil Samples

PA:Fe and PA:Zn MR of initial and 1-year stored samples of unfortified control and fortified lentil samples from three milled LPTs are shown in Table 5. Among the samples from all three LPTs and both batches, unfortified controls had the highest PA:Fe MR which was far higher than the PA:Fe MR of Fe- and dual-fortified samples. Similarly, the highest PA:Zn MR was found in unfortified control samples for all three LPTs in two batches. Although the Zn-fortified lentil had lower PA:Zn MR than control and Fe-fortified samples, the lowest PA:Zn MR was found in dual-fortified lentil. After 1 year of storage, PA:Fe and PA:Zn MR were increased than the initial stage samples with a few exceptions. This could be due to a decrease of Fe and Zn concentration after 1 year of storage of all types of samples with a few exceptions. Overall, considering both PA:Fe and PA:Zn MR, dual-fortified lentil had the lowest PA:Fe and PA:Zn MR compared with other samples.

**Table 5:** PA:Fe and PA:Zn molar ratios of initial (after fortification) and 1-year stored samples of unfortified control, Fe-fortified, Zn-fortified, and dual-fortified dehulled lentil samples from three milled product types of lentil.

**Samples**	**PA:Fe molar ratio**		**PA:Zn molar ratio**	
**Red football**	**Initial**	**After 1 year**	**Initial**	**After 1 year**
Unfortified control (0.5% canola oil)	7.06	7.78	14.61	16.19
ZnSO_4_·H_2_O fortified	6.96	8.14	5.12	7.25
NaFeEDTA fortified	1.70	2.34	11.92	9.30
ZnSO_4_·H_2_O and NaFeEDTA fortified	1.82	2.63	3.55	3.97
**Red split**				
Unfortified control (0.5% canola oil)	9.64	10.66	19.11	21.56
ZnSO_4_·H_2_O fortified	9.33	10.62	6.54	8.96
NaFeEDTA fortified	2.52	3.58	16.40	12.59
ZnSO_4_·H_2_O and NaFeEDTA fortified	2.39	8.02	4.69	17.06
**Yellow split**				
Unfortified control (0.5% canola oil)	13.86	14.35	30.50	33.28
ZnSO_4_·H_2_O fortified	12.99	14.16	8.17	11.35
NaFeEDTA fortified	3.58	3.99	27.05	17.78
ZnSO_4_·H_2_O and NaFeEDTA fortified	2.70	4.00	5.10	5.73

### Correlation Coefficients Between the Four Measured Variables

Correlation coefficients between the four measured variables are presented in Table 6. Significant correlations were observed for (Fe) vs. RFeB%, RFeB% vs. PA:Fe MR, (Fe) vs. PA:Fe MR, and (Zn) vs. PA:Zn MR from all three LPTs in two batches. Overall, a positive correlation was observed only for (Fe) vs. RFeB%, and an inverse relationship was observed for the other three relationships.

**Table 6:** Pearson correlation coefficients for Fe concentration vs. PA:Fe molar ratio, iron (Fe) concentration vs. relative Fe bioavailability (RFeB%), RFeB% vs. PA:Fe molar ratio, and Zn concentration vs. PA:Zn molar ratio of nine dehulled lentil samples of each of the three product types (red football, red split, and yellow split) containing unfortified lentil and fortified lentil.

**Lentil samples**	**(Fe) vs. RFeB%**		**RFeB% vs. PA:Fe molar ratio**		**(Fe) vs. PA:Fe molar ratio**		**(Zn) vs. PA:Zn molar ratio**	
	**Initial**	**After 1 year**	**Initial**	**After 1 year**	**Initial**	**After 1 year**	**Initial**	**After 1 year**
Red football (*n* = 9)	0.892^**^ (0.001)	0.955^**^ (0.001)	−0.866^**^ (0.011)	−0.938^**^ (0.001)	−0.982^**^ (0.001)	−0.977^**^ (0.001)	−0.984^**^ (0.001)	−0.980^**^ (0.001)
Red split (*n* = 9)	0.970^**^ (0.002)	0.983^**^ (0.001)	−0.895^**^ (0.001)	−0.982^**^ (0.001)	−0.953^**^ (0.001)	−0.951^**^ (0.001)	−0.979^**^ (0.001)	−0.978^**^ (0.001)
Yellow split (*n* = 9)	0.971^**^ (0.002)	0.970^**^ (0.002)	−0.822^*^ (0.023)	−0.881^**^ (0.008)	−0.932^**^ (0.002)	−0.921^**^ (0.001)	−0.963^**^ (0.001)	−0.953^**^ (0.001)
All samples (*n* = 27)	0.867^**^ (0.001)	0.913^**^ (0.001)	−0.680^**^ (0.001)	−0.806^**^ (0.001)	−0.899^**^ (0.001)	−0.910^**^ (0.001)	−0.869^**^ (0.001)	−0.892^**^ (0.001)

** *Correlation is significant at the 0.01 level (2-tailed)*.

* *Correlation is significant at the 0.05 level (2-tailed)*.

## Discussion

Lentil is an ancient food crop like wheat and barley. Globally, more than 50 and 120 countries produce and consume lentil, respectively (41). Global lentil consumption is rising much more quickly than other pulses (36), but still lower than the consumption rate for pulses recommended by FAO. Pulses consumed with staple cereals provide complementary protein quality, but the pulse–cereal combination provides insufficient mineral micronutrient nutrition. Many countries consume lentil in their daily meal as a staple or partial staple food. As part of the micronutrient improvement program, a dual-fortification program with both Fe and Zn was initiated with the objective of improving the Fe and Zn concentration and bioavailability in lentil. Like other staple cereals and pulses, lentil also contains PA and polyphenols that inhibit Fe and Zn absorption. Improvement of Fe and Zn concentration and their bioavailability can overcome these limitations. Lentil is highly acceptable as a food that provides inexpensive protein and dietary fiber compared with animal sources, combined with fast cooking time relative to all major pulses. Moreover, recent studies showed that not all polyphenols inhibit Fe and Zn absorption, and some polyphenols can promote Fe and Zn absorption (42, 43). In this study, all three lentil product types were dehulled/milled, and seed coats were removed before fortification. Thus, seed coat polyphenols do not play a role in the Fe bioavailability from these lentil products (38).

The rationale to use three product types to fortify had several reasons. Lentil consumption patterns vary based on cotyledon color, presence or absence of seed coat, availability, tradition, and consumer preference. For example, red cotyledon lentil has wide acceptability in South Asia and the Middle East (44). Yellow cotyledon lentils are mostly consumed in Europe and are also used in several value-added or processed food products (e.g., snacks) worldwide. Some lentil-consuming regions prefer split types, whereas some regions prefer football types. Split lentil has more surface area than red football lentil, and surface area influences fortificant absorption. The nutritional composition of the three lentil types has been included in Supplementary Table 4 (45). Significant differences were also reported for starch, protein, and phenolic compounds concentration between red and yellow lentils (46, 47). This difference might influence mineral concentration and Fe bioavailability. Moreover, our study results also showed different Fe, Zn, and PA concentrations, and relative Fe bioavailability between three product types.

The bioavailability of micronutrients from food is a stepwise process. Initially, Fe is released through digestion, followed by absorption in the circulation system, and finally processed and incorporated into a body's functional compartment (32, 48). Both *in vitro* and *in vivo* models are used for assessing the bioavailability of Fe and Zn. In this study, Fe bioavailability was assessed using an *in vitro* Caco-2 cell bioassay for both unfortified and fortified RF, RS, and YS lentil samples from fresh and stored samples. This model mimics conditions in the small intestine, and ferritin formation in the Caco-2 cell monolayer is considered a marker for Fe intake (35). There are some limitations of Caco-2 cell bioassay, for example, it cannot be a substitute for an *in vivo* model or animal model (35). But considering its high sensitivity, cost-effectiveness, and speed of measurement of Fe availability in foods, the Caco-2 cell bioassay represents a prediction of Fe absorption by humans. Caco-2 cell model results were strongly correlated (*R* = 0.968; *p* < 0.001) with human Fe absorption studies (49) and with human and animal efficacy studies of Fe absorption from biofortified crops (50).

The PA:Zn MR predicts the inhibiting effect of phytate on the Zn bioavailability (51). Phytate:Zn MR of 15:1 is considered a threshold level for Zn absorption, and above this point Zn absorption is greatly reduced, resulting in negative Zn balance, a level considered to be suboptimal for Zn status (29, 52). PA:Zn molar ratios of >15, <10, and <5 are considered as low, medium, and high absorption status of Zn (53). PA concentrations were measured using a colorimetric assay kit for the same samples used for Fe and Zn bioavailability, which has the limitation that it does not assess myo-inositol in either phytase/alkaline phosphatase released form or in the free form (54). This type of analysis is widely used, sometimes providing more accurate and reliable estimates than HPLC, and it is easy to assess compared with HPLC (54–56).

Significant differences were observed between control and fortified lentil samples within two batches for Fe, Zn, and PA concentration and RFeB%. Despite the enhancing Fe fortification dose, the average Fe concentration in lentils slightly increased and not in a proportional manner. It could be due to the diverse product types, absorption capability, the composition of seed, and its interaction with fortificants. Among the three LPTs, sample 6 (Fe fortified) was fortified with 16 mg of Fe, and sample 8 (dual fortified) was fortified with 16 mg of Fe and 8 mg of Zn. With similar amounts of Fe in these two sample types, the addition of 8 mg Zn in the dual-fortified samples provided 1.4, 7.4, and 10.3 mg more Fe at the initial stage (after fortification) in RF, RS, and YS, respectively. Similarly, in all three LPTs, sample 6 (Fe fortified) and sample 9 (dual fortified) were fortified with similar amounts of Fe, but the addition of 12 mg Zn in the dual-fortified one resulted in higher Fe concentration in the dual-fortified sample compared with the Fe-fortified one. A similar trend was observed for samples stored for 1 year. Most of the studies with Zn-fortified foods have reported that the addition of Zn to food did not adversely affect the absorption of other minerals, including Fe (57). In a study in Iran, three groups of Zn-deficient women consumed unfortified, low Zn (50 ppm) fortified, and high Zn (100 ppm) fortified bread, respectively (58). Results showed that the high Zn bread group had a significantly greater absorption of not only Zn but also Fe. Results from this study also showed that RFeB% of RF and YS samples that were fortified with ZnSO_4_·H_2_O were decreased. A reduction of Fe absorption from ZnSO_4_·H_2_O fortified wheat flour dumplings was also reported in a previous study with Indonesian children (59). Again, among the three LPTs of this study, compared with Fe-fortified samples, YS dual-fortified samples had a higher amount of Fe increment than RF and RS samples. This indicates that YS lentil might have better absorption, providing more bioavailable Fe in the dual-fortified state than in the Fe-fortified state. This result can be further evaluated to confirm whether the addition of Zn with Fe would provide more available Fe in *in vitro* digestion.

Most of the fortified (both single and dual-fortified) samples had significantly different PA concentrations compared with the two controls, with a decreasing trend of PA concentration after fortification. The difference in PA concentration was minimal between control and Zn-fortified samples for all three LPTs, indicating that Zn fortification had a comparatively low effect on PA decrement after fortification compared with Fe and dual fortification. PA binds with multivalent cations such as Fe, Zn, Ca, and Mg, forming insoluble complexes that decrease Fe absorption in the small intestine (60). In this study, PA concentration in sample 2 (unfortified and polished control sample) of RF, RS, and YS lentil was 0.63, 0.82, and 0.89 mg 100 g^−1^ of lentil, respectively. In sample 9 (fortified with 24 mg of Fe and 12 mg of Zn 100 g^−1^ of lentil), it was reduced to 0.53, 0.71, and 0.79 mg 100 g^−1^ in RF, RS, and YS lentil, respectively. RFeB% also increased in sample 2 to sample 9 by 91.3 to 307.3%, 113.6 to 521.8%, and 122.0 to 519.5% in RF, RS, and YS lentil samples at the initial stage, respectively. Similar trends were observed for 1-year stored samples for all the three LPTs (Supplementary Tables 1–3). After 1 year of storage, PA concentration was either increased or decreased compared with the initial batch samples and did not show any consistent pattern. It could be a random error from the analysis. Overall, the increase of Fe, Zn, and RFeB% and decrease of PA concentration in dual-fortified lentils compared with controls or single-fortified lentils indicated that dual-fortified lentil samples could provide adequate amounts of Fe and Zn while increasing bioavailability.

With the reduction of PA concentration in fortified lentil compared with unfortified lentil, PA:Fe and PA:Zn MR were reduced significantly in fortified lentil compared with unfortified lentil samples for all three LPTs and from two batches. Both Fe- and dual-fortified lentil samples had lower PA:Fe MR. Zn-fortified and dual-fortified lentil samples had lower PA:Zn MR compared with control samples. Overall, dual-fortified samples had the lowest PA:Fe and PA:Zn MR, indicating that, compared with single fortification, dual fortification would help to reduce both PA:Fe and PA:Zn MR. The reduction of PA concentration was also reported in our previous studies with Fe-fortified lentil studies. During the fortification process, dephytinization reduced PA concentration from 6.2 to 4.6 mg g^−1^ in Fe-fortified lentil (56), thereby reducing PA:Fe molar ratio in Fe-fortified lentil. Another study also reported that dephytinization and fortification reduced PA:Fe MR from 24:1 to 0.3:1 in Fe-fortified fonio (*Digitaria exilis*) meals in West African women (61). Similarly, dephytinization would work for reducing PA:Zn MR in this study. In a review, Gibson et al. (62) revealed that of 27 foods processed and fortified complementary with mineral and vitamins, 25 and 70% had lower PA:Fe and PA:Zn MR, respectively, when refined wheat flour, white rice flour, or rice flakes were used rather than unrefined cereals, oleaginous seeds, and or legumes (62).

Both fortified and unfortified lentil samples had a decrease of Fe and Zn concentration after 1 year of storage from 6.4 to 14.5% and 4.5 to 17.2%, respectively. Fortification may not have an effect on the decrease of Fe and Zn concentration as the decrement was observed for both fortified and control samples in all three LPTs from two batches. Moisture content was measured before sample preparation and was similar for an indication that a decrease of both Fe and Zn concentration after 1 year could be due to the absorption of moisture in second batch samples during sample preparation for bioavailability assessment. The initial fortified samples were analyzed for moisture content and water activity at the Saskatchewan Food Industry Development Center, following the “Official Methods of Analysis of the Association of Official Analytical Chemists (AOAC),” 16th Edition (1995) 42.1.03 (978.18). The moisture content and water activity of unfortified lentils (control) was 9.86% and 0.45, respectively, similar to that of the dual-fortified lentils (10.41% and 0.44). There is evidence for losses of Fe and Zn (<10%) in fortified rice after storage for up to 1 year at 40°C and 75% humidity (63).

WHO has recommended Fe and Zn fortificants and their doses for Fe and Zn fortification in different food products (64). FAO and WHO recommended that the EAR of Fe and Zn is 29.4 and 4.9 mg for males and 18.8 and 7.0 mg for females, respectively (6). Considering Fe, Zn, and PA concentration, RFeB%, PA:Fe MR, and PA:Zn MR, the dual-fortified samples (fortified with 16 mg of Fe and 8 mg of Zn 100 g^−1^ of lentil) can provide approximately 14 mg of Fe and 6.5 mg of Zn from in 50 g of lentil (dry basis)—a major part of the EAR of Fe and Zn currently recommended by WHO. Considering the tolerable upper intake level of Fe (45 mg/person/day) and Zn (40 mg/person/day) for adults (65, 66), 50 g of dual-fortified lentil from sample 9 (24 mg of Fe and 12 mg of Zn 100 g^−1^ of lentil) is considered safe for human consumption.

## Conclusion

Because Fe and Zn are considered the most abundant micronutrient minerals in humans, and have common dietary sources, dual-fortified lentil could be a potential vehicle to provide a significant amount of Fe and Zn to alleviate Fe and Zn deficiency, especially in regions where lentil is frequently consumed as a staple or partial staple food. The stability of added micronutrients in fortified food over time is a key factor that influences the success or failure of the fortification program. Overall results from this study revealed that Fe, Zn, and RFeB% were increased significantly in two batches of samples from three LPTs with the increase of Fe and Zn doses but decreased significantly but numerically negligible after 1 year of storage, indicating a minor effect of fortification on Fe and Zn concentration over time. PA concentration was decreased from 8 to 15% after fortification in all samples of three LPTs from two batches but showed a different pattern of influence after storage indicates a positive effect of fortification on an increment of Fe and Zn bioavailability. Moreover, in both batches, dual-fortified lentil had the lowest PA:Fe and PA:Zn MR compared with other samples. Around 56% of the world's lentil is consumed in Asian countries with tropical to sub-tropical temperature [high temperature (>35°C) and high relative humidity (>85%)] that may have an influence on RFeB%. We have not yet investigated the influence of storage period on the stability of dual-fortified lentil under retail storage conditions in tropical and sub-tropical regions. Future additional research studies will take this into account, for example by investigating (1) choice of suitable packaging relevant to various retail market conditions of lentil consuming regions and (2) an efficacy trial with Fe- and Zn-deficient populations using dual-fortified lentil products.

## Data Availability Statement

The original contributions generated for the study are included in the article/Supplementary Material, further inquiries can be directed to the corresponding author/s.

## Author Contributions

RP, RG, and AV conceived and designed the study. RP analyzed the study and prepared the draft article. All authors reviewed all documents critically and approved the final article for submission to the journal.

## Conflict of Interest

The authors declare that the research was conducted in the absence of any commercial or financial relationships that could be construed as a potential conflict of interest.

## Abbreviations

LPT: lentil product type
RDA: recommended daily allowance
RF: red football
RFeB%: relative iron bioavailability
RS: red split
YS: yellow split
MR: molar ratio.
