# Supplementary material for: Iron- and Zinc-Fortified Lentil (Lens culinaris Medik.) Demonstrate Enhanced and Stable Iron Bioavailability After Storage
Source: Front Nutr. 2021 Jan 8;7:614812. doi: 10.3389/fnut.2020.614812 (PMC7819975; doi:10.3389/fnut.2020.614812)
Supplement: Supplementary file 1 [file Data_Sheet_1.docx]

**Supplementary Figure 1**. Nine dehulled lentil samples (three samples from each of three product types, red football, red split and yellow split) fortified with 16 mg of Fe and 8 mg of Zn.100^-1^ of lentil. Three samples at 1^st^ row are unfortified control, at 2^nd^ row are fortified samples at initial stage and at 3^rd^ row are fortified samples after one year of storage.

**
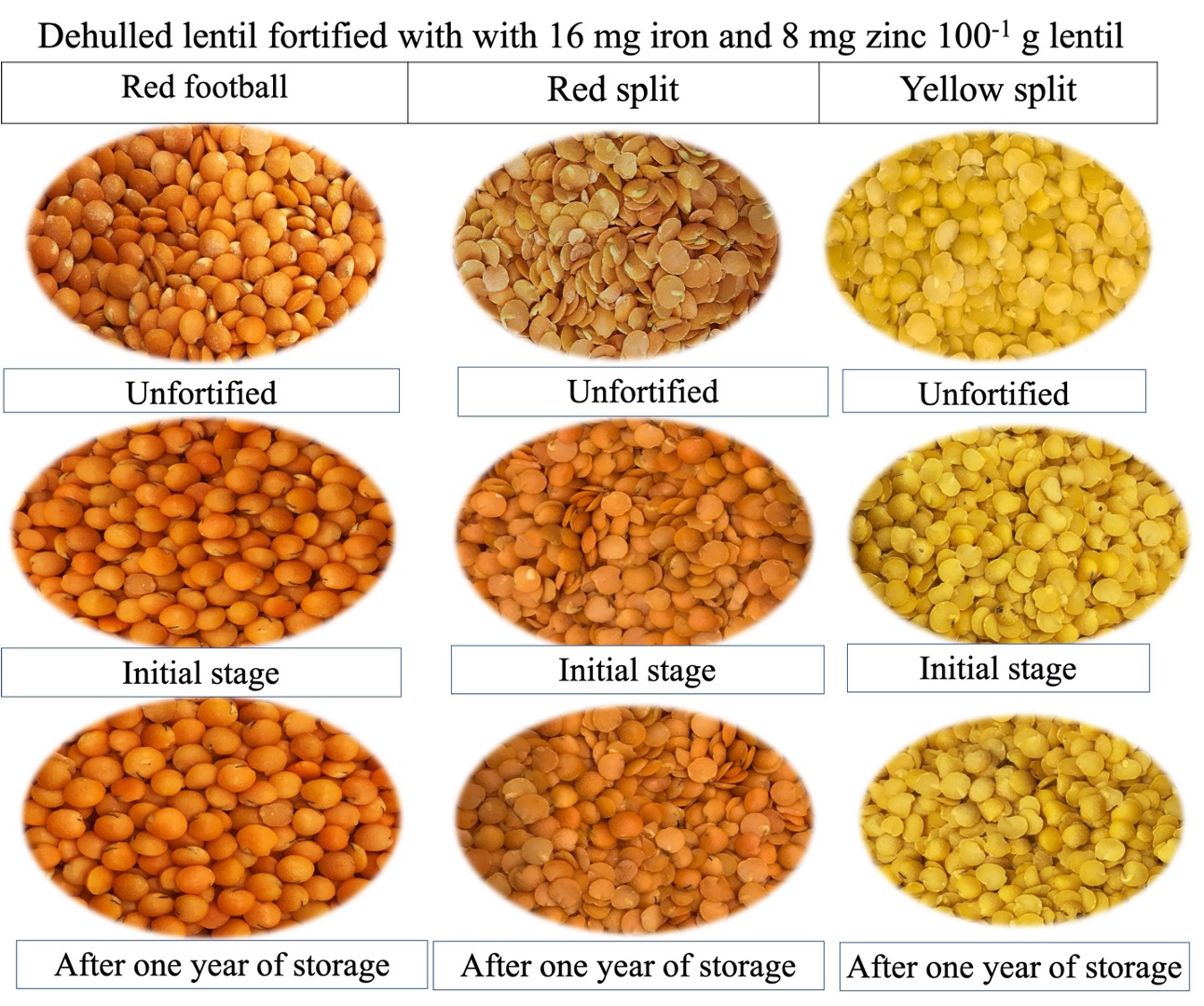
**
