# Supplementary material for: Iron- and Zinc-Fortified Lentil (Lens culinaris Medik.) Demonstrate Enhanced and Stable Iron Bioavailability After Storage
Source: Front Nutr. 2021 Jan 8;7:614812. doi: 10.3389/fnut.2020.614812 (PMC7819975; doi:10.3389/fnut.2020.614812)
Supplement: Supplementary file 2 [file Table_1.DOCX]

**Supplementary Table 1.** “ng ferritin (mg protein)^-1^”, relative Fe bioavailability (RFeB%) and “RFeB (%) increase/decrease than control” of nine dehulled lentil samples of red football, containing unfortified lentil (sample 1-2) and fortified lentil (samples 3-9) assessed using Caco-2 cell bioassay.

| Red football lentil samples | Fortificant dose added  100^-1^ g of lentil | | 1st batch (after fortification) | | | 2nd batch (one-year of storage) | | |
| --- | --- | --- | --- | --- | --- | --- | --- | --- |
|  |  |  | ng ferritin (mg protein)^-1 a^ | RFeB% | %RFeB increase/  decrease than control | ng ferritin (mg protein)^-1 a^ | RFeB% | %RFeB increase/  decrease than control |
|  | Fe (mg) NaFeEDTA | Zn (mg)  ZnSO_4_H_2_O |  |  |  |  |  |  |
| Sample 1 ^b^ | Unfortified and unpolished | | 11.8 ± 0.8 b | 100.0 | 0.0 | 67.1 ± 4.6 b | 100.0 | 0.0 |
| Sample 2 ^c^ | Unfortified and polished | | 10.8 ± 0.3 b | 91.3 | 8.7 | 60.5 ± 2.7 b | 90.1 | -9.9 |
| Sample 3 ^d^ | -- | 6 | 6.3 ± 0.9 a | 53.3 | -46.7 | 50.7 ± 1.8 a | 75.5 | -24.5 |
| Sample 4 ^d^ | -- | 12 | 6.1 ± 0.1 a | 51.7 | -48.3 | 46.8 ± 1.7 a | 69.8 | -30.2 |
| Sample 5 ^e^ | 16 | -- | 17.9 ± 0.5 c | 150.9 | 50.9 | 139.2 ± 7.1 d | 207.4 | 107.4 |
| Sample 6 ^e^ | 24 | -- | 26.0 ± 2.4 d | 220.1 | 120.1 | 148.8 ± 5.4 e | 221.7 | 121.8 |
| Sample 7 ^f^ | 12 | 12 | 19.0 ± 0.7 c | 160.8 | 60.8 | 131.7 ± 2.9 c | 196.2 | 96.2 |
| Sample 8 ^f^ | 16 | 8 | 27.6 ± 2.9 d | 233.1 | 133.1 | 165.1 ± 1.8 f | 246.0 | 146.0 |
| Sample 9 ^f^ | 24 | 12 | 36.4 ± 0.9 e | 307.3 | 207.3 | 198.2 ± 9.2 g | 295.3 | 195.3 |
| Pearson correlation coefficients ^g^ | | | 0.95** | | | | | |

^a^ Mean ± SD. Mean scores for ng ferritin (mg protein)^-1^ followed by different letters within columns are significantly different (p < 0.001). ^b^ Unfortified control lentil; ^c^ Unfortified control but polished with 0.5% canola oil; ^d^ Zn-fortified lentil with ZnSO_4_H_2_O, ^e^ Fe-fortified lentil with NaFeEDTA; ^f^ Dual-fortified lentil with NaFeEDTA and ZnSO_4_H_2_O. ^g^ Pearson correlation coefficients for RFeB% between two batches. **Correlation is significant at the 0.01 level (2-tailed); * Correlation is significant at the 0.05 level (2-tailed).
