# Supplementary material for: Iron- and Zinc-Fortified Lentil (Lens culinaris Medik.) Demonstrate Enhanced and Stable Iron Bioavailability After Storage
Source: Front Nutr. 2021 Jan 8;7:614812. doi: 10.3389/fnut.2020.614812 (PMC7819975; doi:10.3389/fnut.2020.614812)
Supplement: Supplementary file 3 [file Table_2.DOCX]

**Supplementary Table 2**. “ng ferritin (mg protein)^-1^”, relative bioavailability (RFeB%) and “%RFeB increase/decrease than control” of nine dehulled lentil samples of red split, containing unfortified lentil (sample 1-2) and fortified lentil (samples 3-9) assessed using Caco-2 cell bioassay.

| Red split lentil samples | Fortificant dose added  100^-1^ g of lentil | | 1st batch (after fortification) | | | 2nd batch (one-year of storage) | | |
| --- | --- | --- | --- | --- | --- | --- | --- | --- |
|  | Fe (mg) NaFeEDTA | Zn (mg)  ZnSO_4_H_2_O | ng ferritin (mg protein)^-1 a^ | RFeB% | %RFeB increase/  decrease than control | ng ferritin (mg protein)^-1 a^ | RFeB% | %RFeB increase/  decrease than control |
| Sample 1 ^b^ | Unfortified and unpolished | | 7.1 ± 0.7 a | 100.0 | 0.0 | 46.4 ± 0.2 a | 100.0 | 0.0 |
| Sample 2 ^c^ | Unfortified and polished | | 7.9 ± 0.5 a | 113.6 | 13.6 | 46.1 ± 1.4 a | 99.4 | -0.1 |
| Sample 3 ^d^ | -- | 6 | 8.5 ± 0.2 a | 120.6 | 20.6 | 44.7 ± 0.4 a | 96.4 | -3.6 |
| Sample 4 ^d^ | -- | 12 | 8.2 ± 0.9 a | 116.1 | 16.0 | 43.9 ± 1.5 a | 94.6 | -5.4 |
| Sample 5 ^e^ | 16 | -- | 20.3 ± 3.4 b | 288.9 | 188.9 | 142.8 ± 0.6 b | 308.0 | 208.0 |
| Sample 6 ^e^ | 24 | -- | 28.9 ± 0.4 c | 411.1 | 311.0 | 191.3 ± 0.2 c | 412.5 | 312.5 |
| Sample 7 ^f^ | 12 | 12 | 19.0 ± 0.6 b | 270.7 | 170.7 | 155.9 ± 0.7 b | 336.3 | 236.3 |
| Sample 8 ^f^ | 16 | 8 | 32.3 ± 1.6 d | 459.4 | 359.4 | 174.7 ± 1.2 d | 376.8 | 276.8 |
| Sample 9 ^f^ | 24 | 12 | 36.7 ± 2.4 e | 521.8 | 421.8 | 210.0 ± 0.9 e | 452.9 | 353.0 |
| Pearson Correlation ^g^ | | | 0.96** | | | | | |

^a^ Mean ± SD. Mean scores for ng ferritin (mg protein)^-1^ followed by different letters within columns are significantly different (p < 0.001). ^b^ Unfortified control lentil; ^c^ Unfortified control but polished with 0.5% canola oil; ^d^ Zn-fortified lentil with ZnSO_4_H_2_O, ^e^ Fe-fortified lentil with NaFeEDTA; ^f^ Dual-fortified lentil with NaFeEDTA and ZnSO_4_H_2_O. ^g^ Pearson correlation coefficients for RFeB% between two batches. **Correlation is significant at the 0.01 level (2-tailed); * Correlation is significant at the 0.05 level (2-tailed).
