# Supplementary material for: Iron- and Zinc-Fortified Lentil (Lens culinaris Medik.) Demonstrate Enhanced and Stable Iron Bioavailability After Storage
Source: Front Nutr. 2021 Jan 8;7:614812. doi: 10.3389/fnut.2020.614812 (PMC7819975; doi:10.3389/fnut.2020.614812)
Supplement: Supplementary file 4 [file Table_3.DOCX]

**Supplementary Table 3.** “ng ferritin (mg protein)^-1^”, relative bioavailability (RFeB%) and “RFeB (%) increase/decrease than control” of nine dehulled lentil samples of yellow split, containing unfortified lentil (samples 1-2) and fortified lentil (samples 3-9) assessed using Caco-2 cell bioassay.

| Yellow split lentil samples | Fortificant dose added  100^-1^ g of lentil | | 1st batch (after fortification) | | | 2nd batch (one-year of storage) | | |
| --- | --- | --- | --- | --- | --- | --- | --- | --- |
|  | Fe (mg) NaFeEDTA | Zn (mg)  ZnSO_4_H_2_O | ng ferritin (mg protein)^-1 a^ | RFeB% | %RFeB increase/decrease than control | ng ferritin (mg protein)^-1 a^ | RFeB% | %RFeB increase/decrease than control |
| Sample 1 ^b^ | Unfortified and unpolished | | 16.3 ± 2.1 a | 100.0 | 0.00 | 44.9 ± 1.3 a | 100.0 | 0.0 |
| Sample 2 ^c^ | Unfortified and polished | | 19.9 ± 1.7 a | 122.0 | 22.1 | 37.0 ± 0.8 a | 82.4 | -17.6 |
| Sample 3 ^d^ | -- | 6 | 11.1 ± 1.7 a | 68.3 | -31.7 | 30.1 ± 1.5 a | 67.0 | -33.0 |
| Sample 4 ^d^ | -- | 12 | 12.8 ± 2.1 a | 78.1 | -21.8 | 29.2 ± 1.5 a | 65.0 | -35.0 |
| Sample 5 ^e^ | 16 | -- | 33.9 ± 3.1 b | 208.1 | 108.1 | 84.0 ± 6.8 b | 186.3 | 86.9 |
| Sample 6 ^e^ | 24 | -- | 40.1 ± 2.8 c | 245.5 | 145.5 | 96.1 ± 4.6 c | 213.8 | 113.8 |
| Sample 7 ^f^ | 12 | 12 | 37.4 ± 2.3 bc | 229.1 | 129.1 | 132.9 ± 8.3 d | 295.8 | 195.9 |
| Sample 8 ^f^ | 16 | 8 | 70.0 ± 4.4 d | 428.9 | 328.9 | 164.2 ± 3.6 e | 365.3 | 265.3 |
| Sample 9 ^f^ | 24 | 12 | 84.8 ± 7.4 e | 519.5 | 328.94 | 176.7 ± 13.4 f | 393.3 | 293.3 |
| Pearson correlation coefficients ^g^ | | | 0.96** | | | | | |

^a^ Mean ± SD. Mean scores for ng ferritin (mg protein)^-1^ followed by different letters within columns are significantly different (p < 0.001). ^b^ Unfortified control lentil; ^c^ Unfortified control but polished with 0.5% canola oil; ^d^ Zn-fortified lentil with ZnSO_4_H_2_O, ^e^ Fe-fortified lentil with NaFeEDTA; ^f^ Dual-fortified lentil with NaFeEDTA and ZnSO_4_H_2_O. ^g^ Pearson correlation coefficients for RFeB% between two batches. **Correlation is significant at the 0.01 level (2-tailed); * Correlation is significant at the 0.05 level (2-tailed).
