# Supplementary material for: Iron- and Zinc-Fortified Lentil (Lens culinaris Medik.) Demonstrate Enhanced and Stable Iron Bioavailability After Storage
Source: Front Nutr. 2021 Jan 8;7:614812. doi: 10.3389/fnut.2020.614812 (PMC7819975; doi:10.3389/fnut.2020.614812)
Supplement: Supplementary file 5 [file Table_4.DOCX]

**Supplementary Table 4: The nutritional composition of the three types of lentil product types**

| Nutrient | Units | Value (per 100g)^1^ | | |
| --- | --- | --- | --- | --- |
|  |  | Red football | Red split | Yellow split |
| Energy^2^ | Cal | 321 | 320 | 322 |
| Protein | g | 25.3 | 27.4 | 25.4 |
| Fat (total) | g | 1.28 | 1.14 | 1.37 |
| Carbohydrate (total) | g | 62.6 | 60.2 | 62.1 |
| Dietary fibre (total) | g | 21.0 | 20.5 | 20.2 |
| Moisture | g | 9.1 | 8.9 | 8.9 |
| Sodium | mg | 10.2 | 5.7 | 5.3 |
| Potassium | mg | 988 | 1010 | 883 |
| Calcium | mg | 68.1 | 25.8 | 68.1 |
| Iron | mg | 7.5 | 7.1 | 5.9 |
| Phosphorus | mg | 373 | 375 | 314 |
| Magnesium | mg | 111.0 | 84.4 | 98 |
| Zinc | mg | 4.3 | 4.4 | 3.9 |
| Selenium | ppm | 0.9 | 0.9 | 0.8 |
| Folate | mg | 97.0 | 108 | 102 |
| Vitamin A | RE | <20 | <20 | <20 |
| Vitamin C | mg | 5.7 | 5.8 | 8.9 |

^1^ Energy calculated: [(4 Cal/g x g protein) + (4 Cal/g x (g carbohydrates - g total fibre)) + (9 Cal/g x g fat) + (2 Cal/g x g total fibre)

^2^ Nitrogen to Protein Conversion Factor: 6.25
